# Sirolimus application in patients with autoimmune-associated kidney diseases based on mTORC1 activation: a preliminary exploration and future perspectives

**DOI:** 10.3389/fphar.2026.1791188

**Published:** 2026-07-29

**Authors:** Chuanchuan Sun, Li Wan, Hui Gao, Yishan Ding, Zhen Qu, Feng Yu

**Affiliations:** 1 Department of Nephrology, Peking University International Hospital, Beijing, China; 2 School of Public Health, Peking University, Beijing, China; 3 National Institute of Health Data Science, Peking University, Beijing, China; 4 Nephrology Rehabilitation Center, Beijing Zhongguancun Hospital, Beijing, China; 5 Department of Rheumatology and Immunology, Beijing Tsinghua Changgung Hospital, School of Clinical Medicine, Tsinghua Medicine, Tsinghua University, Beijing, China; 6 Department of Endocrinology and Metabolism, Peking University People’s Hospital, Beijing, China

**Keywords:** autoimmune associated kidney diseases, mTORC1, observational studies, precision therapy, sirolimus

## Abstract

**Background:**

The mTORC1 pathway drives pathogenesis in autoimmune kidney diseases. We hypothesized that intrarenal mTORC1 activation could identify patients likely to benefit from sirolimus, irrespective of their specific diagnosis.

**Methods:**

In this prospective proof-of-concept series, we screened eight patients with active autoimmune kidney disease for mTORC1 activation by immunohistochemistry on renal biopsy. Five positive patients (staining intensity: + to +++) received sirolimus, presenting with lupus nephritis atypical hemolytic uremic syndrome (aHUS), thrombotic microangiopathy (TMA), or IgA nephropathy (IgAN) associated with lung cancer.

**Results:**

Over a mean follow-up of 25 months, four patients exhibited improved or stable renal function. All responders demonstrated moderate-to-high (++ to +++) mTORC1 staining, whereas the single non-responder who progressed to end-stage renal disease had only weak (+) staining. Proteinuria declined to <1 g/day in most responders. Sirolimus was well-tolerated. In two patients with concurrent malignancy, sirolimus did not aggravate stable breast cancer but was discontinued due to lung adenocarcinoma progression.

**Conclusion:**

This study indicates that intrarenal mTORC1 activation levels may help identify patients more likely to respond to sirolimus across various autoimmune kidney diseases, warranting further investigation as a precision-medicine approach.

## 1 Introduction

The mammalian target of rapamycin (mTOR) is a serine/threonine protein kinase originally discovered in the 1970s and now recognized as a central regulator of cell growth, metabolism, proliferation, and survival (Gui and Dai, 2020). Its inhibitor, sirolimus, has demonstrated potent antifungal, immunosuppressive, and antitumor properties (Yoo et al., 2017; Yang et al., 2013). In the kidney, mTOR signaling governs a wide range of physiological and pathological processes, including organ homeostasis, metabolism, regeneration, and transplant rejection (Wu et al., 2021). Accumulating evidence has implicated mTOR pathway activation in the pathogenesis of diverse renal diseases, such as cyst expansion in autosomal dominant polycystic kidney disease (ADPKD), renal cell carcinoma, and immune-mediated disorders like lupus nephritis (LN) (Huynh et al., 2023; Fantus et al., 2016). Despite this compelling mechanistic rationale, the clinical efficacy of sirolimus has been inconsistent across these conditions. For example, sirolimus failed to slow cyst growth in ADPKD, while mTOR inhibitors have shown survival benefits in advanced renal cell carcinoma. In kidney transplantation, mTOR inhibitor-based regimens reduce viral infections and malignancy risk but may be associated with higher rates of acute rejection (Flechner, 2018; Piranavan and Perl, 2021; Perl, 2016; Walz et al., 2010; Serra et al., 2010; Fázio et al., 2023; Pal and Quinn, 2013; Choueiri et al., 2016). Within this landscape, the therapeutic potential of sirolimus in autoimmune-associated kidney diseases—characterized by immune complex deposition and chronic inflammation—remains particularly underexplored (Salet et al., 2023; Dufour et al., 2020; Yap et al., 2012; Cruzado et al., 2011; Kanunnikov et al., 2021).

Glomerulonephritis represents the second most common cause of end-stage renal disease and encompasses a heterogeneous spectrum of disorders unified by immune-mediated injury to the glomerular tuft (Segelmark and Hellmark, 2010). Dysregulation of both innate and adaptive immunity drives the pathogenesis of IgA nephropathy (IgAN), LN, and related conditions. Notably, mTORC1 hyperactivation appears to act as a convergent pathogenic node across these entities. In LN, the degree of intrarenal mTORC1 activation correlates closely with disease activity and histological severity (Perl, 2016; Yap et al., 2012). In antiphospholipid syndrome nephropathy, marked mTORC1 pathway activation within the vasculature has been demonstrated, and sirolimus treatment led to regression of vascular lesions on repeat biopsy (Dufour et al., 2020; Canaud et al., 2014). Critically, the clinical benefit of mTOR inhibition in autoimmunity may depend on the presence of mTORC1 overactivation. A landmark open-label trial of sirolimus in unselected patients with active systemic lupus erythematosus demonstrated only modest efficacy (Lai et al., 2018), whereas studies that stratified patients by mTORC1 activation status—either in renal tissue or functional assays—observed substantial clinical improvement (Canaud et al., 2014; Gao et al., 2023). These divergent results raise the hypothesis that mTORC1 activation status could serve as a precision-medicine approach to sirolimus therapy in autoimmune kidney diseases.

To test this concept, we conducted a prospective case series in which patients with autoimmune kidney disease were screened for mTORC1 activation by immunohistochemistry on renal biopsy. Only those with evidence of pathway activation (positive p-S6RP staining) received sirolimus. This exploratory study evaluated the therapeutic efficacy of sirolimus in this patient population.

## 2 Methods

### 2.1 Patient selection and study design

**FIGURE 1:**
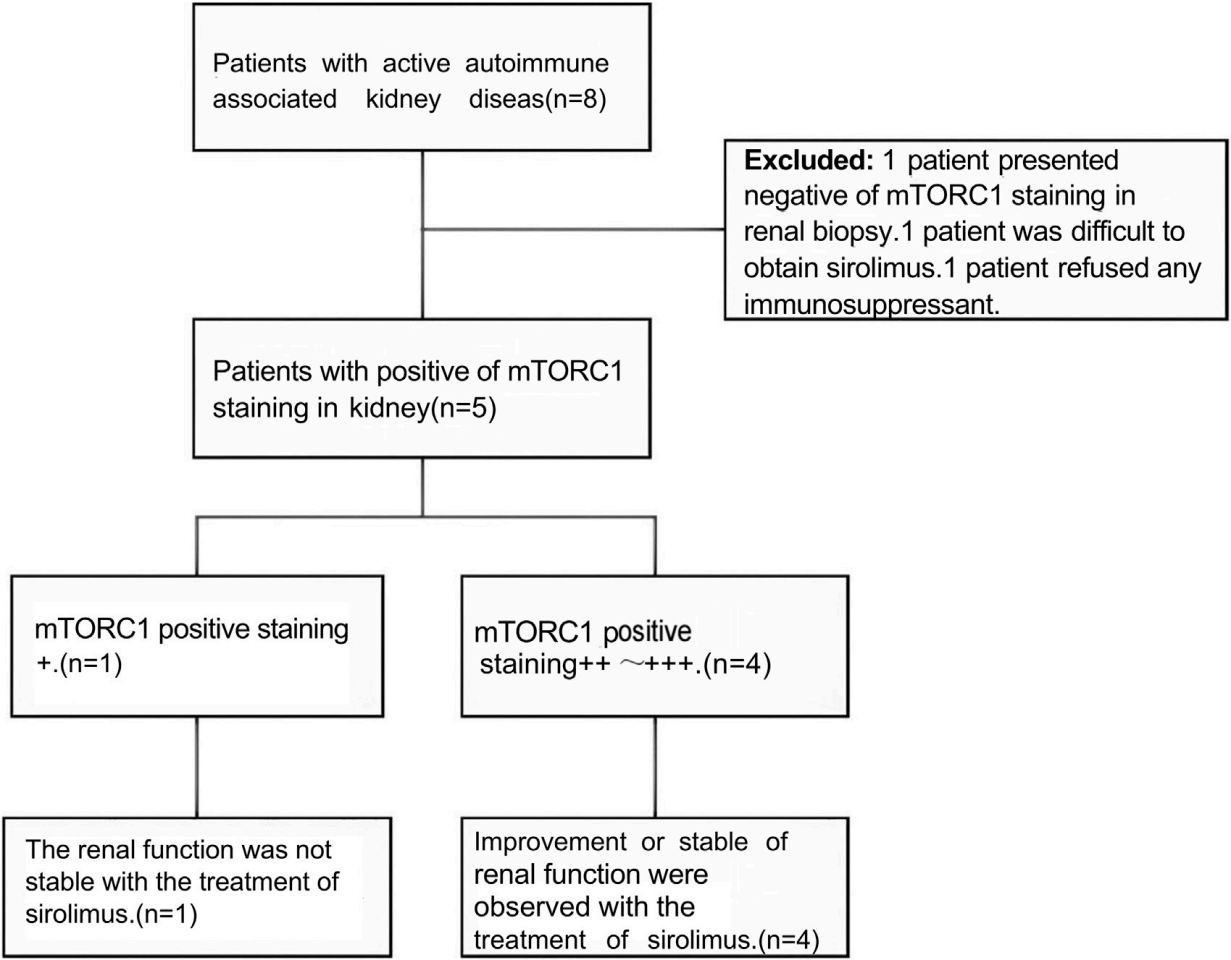
Flow chart of the study sample screening process. mTORC1, mechanistic target of rapamycin complex 1.

Between January 2020 and January 2022, we prospectively screened eight patients with active autoimmune-associated kidney disease at Peking University International Hospital for eligibility based on mTORC1 activity in renal tissue. Three patients were excluded: one due to negative mTORC1 staining on renal biopsy, one who faced challenges in procuring sirolimus, and one who declined any immunosuppressant therapy. Consequently, five patients with positive renal mTORC1 staining were enrolled and initiated on sirolimus treatment. Written informed consent was obtained from all participants, and the study protocol adhered to the principles of the Declaration of Helsinki. The patient selection process is delineated in Figure 1.

### 2.2 Renal pathological assessment and mTORC1 staining

Comprehensive renal pathological evaluation, including light microscopy (hematoxylin and eosin, periodic acid-silver methenamine, periodic acid-Schiff, and Masson’s trichrome staining), direct immunofluorescence (on frozen sections for IgG, IgA, IgM, C3, C1q, fibrinogen, albumin, κ and λ light chains), and electron microscopy, was performed at the Department of Nephrology, Peking University First Hospital. To assess mTORC1 pathway activation, immunohistochemical staining for phosphorylated S6 ribosomal protein (p-S6RP; Rabbit monoclonal antibody, Cell Signaling Technology, USA) was conducted on paraffin-embedded tissue sections (Ding et al., 2023). P-S6RP staining was evaluated using a semi-quantitative system that combined staining intensity (0, negative; 1+, weak; 2+, moderate; 3+, strong) and the percentage of positive cells (<25%, 25%–49%, 50%–74%, ≥75%). The final mTORC1 activation grade was based on the integrated score: + (weak), ++ (moderate), or +++ (strong). All sections were independently scored by two renal pathologists blinded to clinical outcomes, with discrepancies resolved by consensus. Activation was systematically assessed in glomerular, tubular, and vascular compartments.

### 2.3 Treatment protocol and data collection

Enrolled patients received oral sirolimus at a daily dose of 0.5–1.5 mg, with dose adjustments made to maintain a target trough concentration of 5–10 ng/mL. Clinical and laboratory parameters, including blood pressure, 24-h urinary protein excretion, serum creatinine, estimated glomerular filtration rate (eGFR), complete blood count, electrolytes, glucose, cholesterol, and triglycerides, were recorded at baseline and during follow-up. Patients were monitored for adverse events, particularly infections and new-onset neoplastic diseases. Prior to sirolimus initiation, patients had received various background therapies, including angiotensin receptor blockers (ARBs; n = 4), glucocorticoids (n = 4), mycophenolate mofetil (MMF; n = 2), rituximab (RTX; n = 1), plasma exchange (PEX; n = 1), N-acetylcysteine (NAC; n = 1), and sodium-glucose cotransporter 2 inhibitors (SGLT2i; n = 1).

## 3 Results

### 3.1 Patient characteristics

**FIGURE 2:**
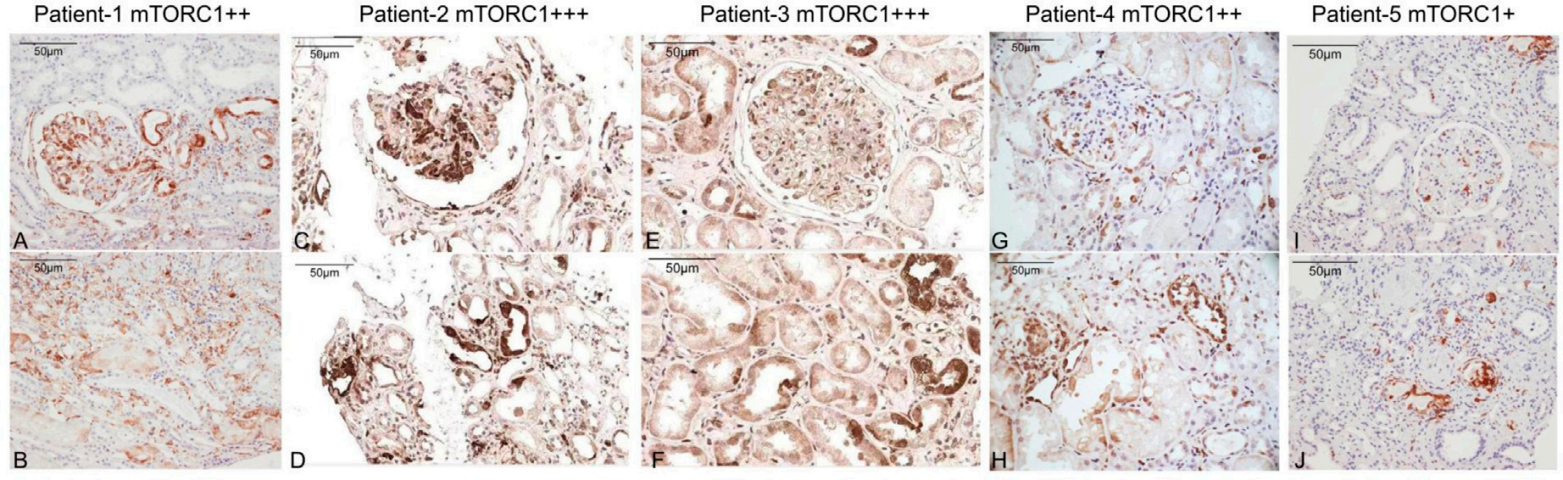
The mTORC1 staining in renal biopsy sections. **(A,B)** Representative renal mTORC1 immunostaining in Patient 1. **(C,D)** Renal mTORC1 immunostaining in Patient 2. **(E,F)** Renal mTORC1 immunostaining in Patient 3. **(G,H)** Renal mTORC1 immunostaining in Patient 4. **(I,J**) Renal mTORC1 immunostaining in Patient 5. Immunohistochemistry staining of p-RPS6 (ser235/236) (surrogate marker of mTORC1 activation) in renal biopsy specimens in these five patients (×200). Patient 1 had aHUS, and presented TMA and ATN in renal biopsy, with 2+ positive of mTORC1 staining. Patient 2 presented nephritis and acute kidney injury during treatment of lung cancer, renal biopsy identified IgA nephritis (Oxford classification as M0E1S0T0C1). Patient 3 had malignant hypertension, diabetes and decreased renal function, renal biopsy presented TMA lesion, diabetic nephropathy (DN) and ischemic glomerular sclerosis, with mTORC1 staining +++. Patients 4 had lupus nephritis - IV (LN-IV) with mTORC1 ++, and patient 5 had LN-III + V and TMA, and presented mTORC1 +. aHUS, atypical hemolytic uremic syndrome; TMA, thrombotic microangiopathy; ATN, acute tubular necrosis; mTORC1, mechanistic target of rapamycin complex 1; IgA, immunoglobulin A; DN, diabetic nephropathy; LN, lupus nephritis.

The baseline demographic, clinical, and pathological characteristics of the five enrolled patients are summarized in Table 1 and Figure 2. The cohort comprised two patients with LN, with the remaining diagnoses including aHUS, TMA, and lung cancer-associated IgAN. Three patients were female, with an age range of 22–73 years. Baseline serum creatinine levels varied from 0.88 to 5.09 mg/dL, and 24-h urinary protein excretion ranged from 1.6 to 4.9 g. The duration of kidney disease prior to sirolimus initiation spanned from 3 months to 24 years, and the interval between renal biopsy and sirolimus treatment commencement ranged from 2 weeks to 4 months. The mean duration of sirolimus therapy was 25 months (range: 3–48 months).

**TABLE 1:** Baseline demographic, clinical and pathologic characteristics.

Clinical parameters	Patient 1	Patient 2	Patient 3	Patient 4	Patient 5
Gender	F	M	M	F	F
Age	25	61	73	36	22
Clinical diagnosis	aHUS	Lung adenocarcinoma nephritis	Hypertension Diabetes	Lupus nephritis Breast Cancer	Lupus nephritis
Renal biopsy	TMA ATN	IgAN (M0E1S0T0C1)	Renal TMA DN	LN-IV	LN-III + V TMA
mTORC1 staining	++	+++	+++	++	+
Creatinine* (mg/dl)	2.26	2.30	1.63	0.88	5.09
eGFR* (ml/min/1.73 m^2^)	28.09	29.6	40.52	84.5	10.9
Urine protein* (g/24 h)	1.6	1.65	4.27	4.9	1.05
Therapy prior to sirolimus	High-dose steroids RTX, PEX, ARB	High-dose steroids ARB	ARB, SGLT2i, NAC	Pulse-dosed steroids MMF, ARB	Pulse-dosed steroids MMF, HCQ, ARB
Prophylactic anti-infection	Sulfonamide	No	No	No	Sulfonamide
Duration from onset to initiation of sirolimus	24 years	4 months	5 years	3 months	4 years
Duration from renal biopsy to initiation of sirolimus	4 months	3 months	1 month	1 month	2 weeks
Time of sirolimus treatment (months)	48	7	31	36	3
Time of Follow-up (months)	48	10	31	36	3
Outcome	CKD 3b, stable	CKD 3a, stable	CKD 3b, stable	CKD 2, stable	CKD 5, dialysis

Abbreviation: aHUS, atypical hemolytic uremic syndrome; TMA: thrombotic microangiopathy; IgAN, IgA nephropathy; LN, lupus nephritis; DN, diabetic nephropathy; ATN: acute tubular necrosis; CKD: chronic kidney disease; eGFR: estimated glomerular filtration rate [CKD-Epidemiology Collaboration (EPI) creatinine equation]; ARB: angiotensin receptor blocker; HCQ: hydroxychloroquine, MMF: mycophenolate mofetil; NAC: N-acetylcysteine; PEX: plasma exchange; RTX: rituximab, SGLT2i: sodium-glucose cotransporter 2 inhibitor.

* At initiation of sirolimus therapy.

### 3.2 Treatment response and outcomes

**FIGURE 3:**
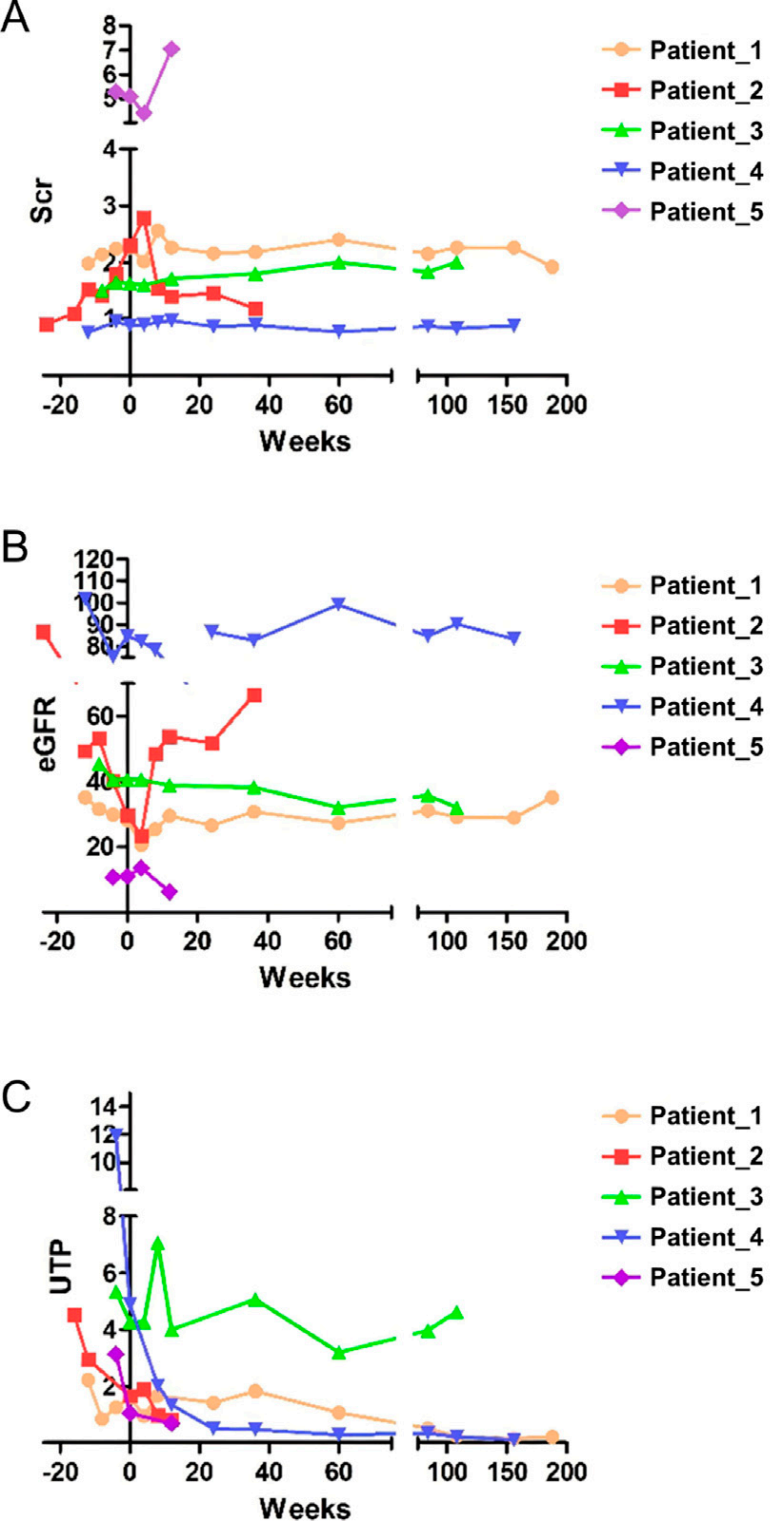
Evaluation of kidney function before sirolimus treatment and during the Follow-up. **(A)** Serial changes in Scr(μmol/L). **(B)** Serial changes in eGFR (ml/min.1.73 m^2^). **(C)** Serial changes in UTP (g/day). Improvement or stable of renal function (Scr and eGFR) were observed in most patients, except Patient_5, who with CKD 5 at baseline started hemodialysis after 3 months. The 24h urine protein (UTP) decreased to <1 g/d for most patients, except Patient_3, whose renal pathological diagnosis was renal TMA, as well as diabetic nephropathy. Scr, serum creatinine; eGFR, estimated glomerular filtration rate; UTP, urinary total protein; CKD, chronic kidney disease.

The individual patient follow-up and outcome data were detailed in Figure 3. Renal function improved or remained stable in the majority of patients. A notable exception was Patient 5, who had baseline stage 5 chronic kidney disease (CKD) and commenced hemodialysis after 3 months of therapy. Interestingly, this was the only patient exhibiting only weakly positive (+) mTORC1 staining; the remaining four patients demonstrated moderate to strong (++ to +++) staining. A reduction in 24-h urinary protein to below 1 g/day was achieved in most patients. Patient 3, whose renal pathology indicated TMA concurrent with diabetic nephropathy, constituted an exception to this trend.

Regarding the two patients with comorbid malignancies, sirolimus was discontinued in Patient 2 (lung adenocarcinoma-associated nephritis) due to oncological progression. In contrast, the breast cancer in Patient 4 (LN) remained stable throughout the sirolimus treatment period.

### 3.3 Safety

Severe Infection was defined as a condition in which pathogens—including bacteria, viruses, and fungi—invade the host, triggering a systemic inflammatory response that leads to organ dysfunction or a life-threatening state. No serious clinical adverse events (e.g., severe infections, new-onset malignancies, oral ulcers, rash, hyperlipidemia, hyperglycemia, myelosuppression, hepatic dysfunction, and tumor recurrence or metastasis) were reported during the follow-up period. Specifically, no incidents of serious infection or *de novo* malignancy were observed. Hematological and biochemical parameters, including white blood cells, hemoglobin, platelets, lactate dehydrogenase, potassium, phosphorus, glucose, cholesterol, and triglycerides, remained largely stable. Patient 3 developed anemia, which was considered potentially related to bone metastases and concomitant chemotherapy rather than to sirolimus therapy.

## 4 Discussion

### 4.1 mTORC1 activation: a common pathological hub in autoimmune-associated kidney diseases

Accumulating evidence indicates that aberrant activation of the mTORC1 signaling pathway serves as a common molecular nexus linking various forms of autoimmune-related renal injury. In LN, our previous studies, corroborated by other reports, have consistently demonstrated a close correlation between the level of intrarenal mTORC1 activation and both disease activity and severity (Mao et al., 2022). Similarly, in antiphospholipid syndrome (APS) nephropathy, the characteristic vascular pathology is associated with marked activation of the mTORC1 pathway within endothelial cells (Dufour et al., 2020; Canaud et al., 2014).

Furthermore, a role for the mTOR pathway has been observed in other conditions such as post-transplant thrombotic microangiopathy (TMA) (Kanunnikov et al., 2021). A summary of recently published studies on mTOR activation in autoimmune-related kidney diseases is provided in Table 1. This widespread activation of mTOR across diverse immune-mediated disorders suggests that mTORC1 may function as a common pathogenic “node,” transcending traditional disease classifications. The underlying mechanisms are likely multifactorial: within immune cells, mTORC1 drives aberrant activation and the release of inflammatory cytokines; in renal parenchymal cells, such as podocytes and tubular epithelial cells, its dysregulation leads to cellular dysfunction, apoptosis, and increased vulnerability to injury; concurrently, it promotes pathological fibrotic processes. Collectively, these actions exacerbate renal inflammation, damage, and ultimately, loss of function (Perl, 2016; Mao et al., 2022; Song et al., 2020; Fan et al., 2023; Shillingford et al., 2006; Brakemeier et al., 2017).

### 4.2 mTOR inhibitor therapy: from empirical to precision-based exploration

Building upon this mechanistic rationale, the mTOR inhibitor sirolimus has been investigated as a potential therapeutic option in various autoimmune kidney diseases, as summarized in Table 2. Numerous case studies provide encouraging evidence: in APS nephropathy, sirolimus treatment not only improved renal function but also demonstrated regression of vascular lesions and inhibition of the mTOR pathway on repeat renal biopsy (Dufour et al., 2020; Canaud et al., 2014). In refractory LN, transplant-associated TMA, and IgAN, sirolimus has also shown potential in stabilizing renal function, reducing proteinuria, and improving survival (Kanunnikov et al., 2021; Yap et al., 2018; Noguchi et al., 2020). Our study represents a continuation and refinement of this therapeutic strategy. In this case series, we prospectively screened patients based on the status of intrarenal mTORC1 activation and administered sirolimus accordingly. The results showed that among the five mTORC1-positive patients, four (with mTORC1 staining intensities of ++ to +++) exhibited improved or stabilized renal function along with reduced proteinuria. However, patient 5 showed weak mTORC1 staining, with a baseline eGFR of approximately 10.9 mL/min/1.73 m^2^. Renal biopsy demonstrated focal proliferative and sclerosing lupus nephritis with superimposed membranous lupus nephritis and chronic tubulointerstitial changes (class III + V, AI = 4, CI = 8). The lack of treatment response in this patient may therefore be attributable to two factors: insufficient mTORC1 activation to drive a meaningful therapeutic benefit from sirolimus, and irreversible chronic renal parenchymal injury that precluded functional recovery even with effective mTORC1 pathway inhibition (Ruiz-Ortega et al., 2020). This finding aligns with conclusions from basic research indicating that mTOR is essential for maintaining cellular function under physiological conditions, and its complete inhibition (e.g., in podocytes or tubular cells) can conversely lead to proteinuria or defects in urinary concentrating ability (Kam et al., 2022; Lovric et al., 2011; Estrada et al., 2019). Consequently, our findings support a precision medicine paradigm in which mTORC1 hyperactivation—serves as the central criterion for sirolimus initiation and helps identify patients most likely to derive therapeutic benefit.

**TABLE 2:** Cases describing mTORi treatment for autoimmune diseases and autoimmune associated kidney diseases.

Author	Case descriptions	mTOR activation	mTORi therapeutic effect
(Canaud et al., 2014)	37 APS nephropathy 10 sirolimus vs. 27 calcineurin inhibitors	Activation of mTORC in the vessels of autopsy specimens	No recurrence of vascular lesions and decreased vascular proliferation on biopsy in sirolimus group
(Dufour et al., 2020)	1 patient with active APS nephropathy. Sirolimus	p-S6RP and p-Ser473 of AKT, AKT/mTORC pathway activation in endothelial cells lining damaged renal vessels	Improvement in glomerular filtration and the inactivation of the AKT/mTORC pathway in endothelial cells in sirolimus group
(Eriksson et al., 2017)	27 patients with SLE sirolimus	/	Increased C4, stable CRP and ESR, decreased PGA and corticosteroid dosage
(Gao et al., 2023)	8 RPF patients Prednisone + sirolimus	mTORC1 pathway (p-S6K) is highly activated	Fibrous tissue reduced by ∼50%; acute inflammation markers decreased by 70%; renal function normalized in most patients. Fibrosis-related T-cell subsets (TH2, TH17, TFH) declined, and TNF and related cytokines restored to healthy levels
(Lai et al., 2018)	29 SLE sirolimus	/	After 12 months, sirolimus expanded CD4^+^CD25+FoxP3+ Tregs and CD8^+^ memory T cells, and inhibited IL-4 and IL-17 production; CD8^+^ memory T cells were selectively expanded in SRI responders. Liver function and lymphocyte counts remained unchanged
(Kanunnikov et al., 2021)	45 transplant-associated TMA sirolimus vs. steroids	​	Better 1-year overall survival and faster time to normalization of LDH in sirolimus group
(Yoon, 2017)	56 patients: 26 RA, 7 PsA, 4 SLE, 3 SSc, 2 anti-Jo-1 syndrome, 2 SpA, 1 MCTD, and 1 vasculitis 28 sirolimus, 28 everolimus	/	Among 26 RA patients, 16 had reduced ESR and CRP, with amelioration or resolution of RA flare. Of 3 patients with skin involvement, one SLE with extensive discoid lupus improved on everolimus, and one cutaneous lupus improved on sirolimus
(Peng et al., 2017)	49 SLE sirolimus	/	SLEDAI-2K decreased, prednisone tapered, and serological indices improved. Remission rates: arthritis 100%, rash 88.8%, thrombocytopenia 46.2%; 41.2% of LN patients achieved renal remission
(Piranavan and Perl, 2021)	73 SLE: 12 LN, 61 without LN sirolimus	/	In the LN cohort, urine protein creatinine ratio, hematuria, anti-DNA antibody levels, and mean steroid dose decreased
(Yap et al., 2018)	16 LN: 5 received sirolimus during active disease vs. 11 received sirolimus during disease quiescence	/	5 patients (sirolimus + prednisolone induction): improved proteinuria, anti-dsDNA, C3. 11 patients (sirolimus + low-dose prednisolone maintenance): continued C3 improvement, stable renal function and proteinuria
(Zhang et al., 2017)	1 LN with APS and AIHA refractory to CTX and MMF. sirolimus	S6RP and Ser473 were activated in renal vascular endothelial cells that expressed CD105	24hUP resolved (<0.5 g/d) after 6 months; LN remained in remission on low-dose prednisone (lowest 2.5 mg/d) for 3.5 years after sirolimus discontinuation
(Cruzado et al., 2011)	23 IgA nephropathy 14 sirolimus vs. 9 enalapril	/	Primary endpoint (blood pressure, proteinuria, hematuria) improved significantly at 12 months in the sirolimus group; isotopic GFR improved and mesangial and endocapillary proliferation reduced
(Noguchi et al., 2020)	135 biopsy-proven IgA nephropathy on native kidneys who underwent KT 107 MMF vs. 28 everolimus	/	Recurrence of IgA nephropathy reduced; everolimus for *de novo* KT was an independent predictor of recurrence-free survival

Abbreviation: APS, antiphospholipid syndrome; SLE, systemic lupus erythematosus; RA,rheumatoid arthritis; SSc, systemic sclerosis; RPF, retroperitoneal fibrosis; TMA, thrombotic microangiopathy; PsA, psoriatic arthritis; SpA, spondyloarthropathy; MCTD, mixed connective tissue disease; LN, lupus nephritis; PSL, Prednisolone; AIHA, autoimmune hemolytic anemia; CTX, cyclophosphamide; MMF, mycophenolat; KT, kidney transplantation; PGA, patient global assessment; SRI, the systemic lupus erythematosus responder index.

In conclusion, our findings implicate mTORC1 hyperactivation as a shared pathogenic mechanism and may help identify patients more likely to benefit from sirolimus therapy. This study is limited by its small sample size, potential confounding from prior background therapies (glucocorticoids, ARBs, MMF, or rituximab), wide disparities in baseline renal function (eGFR ranging from 10.9 to 84.5 ml/min/1.73 m^2^), inconsistent durations of sirolimus therapy and the possibility that age-related cellular senescence and basal mTOR signaling changes amplify mTORC1 activation in the disease state (Chanvillard et al., 2025). Larger prospective studies and non-invasive biomarkers are required to validate the therapeutic potential of mTORC1-directed strategies.

## What is known about this subject

- The mTORC1 signaling pathway has been identified as being aberrantly activated in the pathogenesis of various autoimmune-associated kidney diseases, such as LN and APS nephropathy.
- The mTOR inhibitor sirolimus has shown potential therapeutic effects in some case reports or small-scale studies for specific nephropathies, including refractory lupus nephritis and post-transplant thrombotic microangiopathy.

## What this study adds

- We report the prospective implementation of a strategy that uses intrarenal mTORC1 activation status as a biomarker to guide sirolimus therapy.
- Our preliminary data indicate that the magnitude of mTORC1 activation may help identify patients more likely to benefit from sirolimus therapy.
- Applied to a small cohort of diverse autoimmune nephropathies, this biomarker-guided approach provides early evidence for targeting of shared pathogenic mechanisms.

## Data Availability

The raw data supporting the conclusions of this article will be made available by the authors, without undue reservation.
