# Supplementary material for: Sirolimus application in patients with autoimmune-associated kidney diseases based on mTORC1 activation: a preliminary exploration and future perspectives
Source: Front Pharmacol. 2026 Jul 29;17:1791188. doi: 10.3389/fphar.2026.1791188 (PMC13462518; doi:10.3389/fphar.2026.1791188)
Supplement: Supplementary file 1 [file Supplementaryfile1.docx]

**Supplementary Materials**

**Figure 1. Medication timeline for Patient 1(Date of renal biopsy: 4 February 2020).**RTX, rituximab; PEX, plasma exchange; ARB, angiotensin receptor blocker; MMF, mycophenolate mofetil; SRL, sirolimus.

**Figure 2. Chronological pharmacotherapy timeline for Patient 2(Date of renal biopsy: 1 February 2021).** PD-1, programmed cell death protein 1; Pred, prednisone; MP, methylprednisolone; IgAN, immunoglobulin A nephropathy; SRL, sirolimus.

**Figure 3. Treatment timeline for Patient 3(Date of renal biopsy: 6 April 2021).** ARB, angiotensin ii receptor blocker; SGLT2I, sodium-glucose co-transporter 2 inhibitor; SRL, sirolimus.

**Figure 4. Treatment chronology of Patient 4(Date of renal biopsy: 24 June 2021).** Pred, prednisone; MP, methylprednisolone; SRL, sirolimus.

**Figure 5. Treatment chronology of Patient 5(Date of renal biopsy: 20 August 2020).** IJV Cath for HD, internal jugular vein catheter for hemodialysis; MP, methylprednisolone; SRL, sirolimus; HD, hemodialysis; HDF, hemodiafiltration; HP, hemoperfusion; RTX, Rituximab; Pred, Prednisone; MMF, Mycophenolate Mofetil; HCQ, Hydroxychloroquine.
